# Artificial Teeth

**Published:** 1875-09

**Authors:** 


ARTIFICIAL TEETH.—Persons who are compelled to wear artificial teeth should always remove them at night. It is far better for the mouth that the portion of the membranes which are covered in the daytime should be left uncovered at night. It is possible to keep artificial dentures reasonably sweet and clean by depositing them in suitable disinfecting mixtures during the period allotted to sleep. Perhaps the best and cheapest disinfectant is permanganate of potash in water.

Several persons have swallowed partial sets of artificial teeth, in sleep, and a singular case of this sort now comes to us from the far west. A man named Thompson, living a few miles out of Fresno, Cal., is said to have lost a set of false teeth very mysteriously in 1865, and given them up as stolen. About three years ago, as a local paper tells the story, he caught a severe cold, and since then has been troubled with a painful cough, accompanied by frequent hemorrhage, and it was feared he was a victim of consumption. His physicians having pronounced his case incurable, he travelled for some time, and finally settled down to die. A few months since, however, in one of his violent fits of coughing, he ejected from his throat several pieces of a bony substance. The next day some more were thrown out, and then came a bit of shiny metal. His medical advisers were again summoned, and with their assistance he succeeded in relieving himself of the remaining fragments of his set of teeth, which it now appears he drew into his windpipe during sleep nearly a decade ago, and has carried about within him ever since.

---

CATARRH.—We have known some severe cases of nasal catarrh cured by the daily use of a pinch of snuff, made by scraping to a fine powder, castile soap, and drawing it up the nose with great force.

